# Thresholds and Trade-Offs: Fire Severity Modulates Soil Microbial Biomass-Function Coupling in Taiga Forests, Northeast of China

**DOI:** 10.3390/microorganisms13061318

**Published:** 2025-06-05

**Authors:** Huijiao Qu, Siyu Jiang, Zhichao Cheng, Dan Wei, Libin Yang, Jia Zhou

**Affiliations:** 1 School of Geographical Sciences, Harbin Normal University, Harbin 150025, China; 15045728093@163.com (H.Q.); 13363960039@163.com (S.J.); 2 Key Laboratory of Biodiversity, Institute of Natural Resources and Ecology, Heilongjiang Academy of Sciences, Harbin 150040, China; chengzc928@163.com (Z.C.); weidan_0929@163.com (D.W.); 3 Heilongjiang Huzhong National Nature Reserve, Huzhong 165038, China

**Keywords:** phospholipid fatty acid, microbial functional diversity, soil enzyme activities, *Larix gmelinii* forest, fire sites

## Abstract

Forest fires critically disrupt soil ecosystems by altering physicochemical properties and microbial structure-function dynamics. This study assessed short-term impacts of fire intensities (light/moderate/heavy) on microbial communities in *Larix gmelinii* forests one year post-fire. Using phospholipid fatty acid (PLFA) and Biolog EcoPlate analyses, we found the following: (1) fire reduced soil organic carbon (SOC), dissolved organic carbon (DOC), total nitrogen (TN), and available nitrogen/potassium (AN/AK) via pyrolytic carbon release, while heavy-intensity fires enriched available phosphorus (AP), AN, and AK through ash deposition. (2) Thermal mortality and nutrient-pH-moisture stress persistently suppressed microbial biomass and metabolic activity. Moderate fires increased taxonomic richness but reduced functional diversity, confirming “functional redundancy.” (3) Neither soil microbial biomass nor metabolic activity at the fire site reached pre-fire levels after one year of recovery. Our findings advance post-fire soil restoration frameworks and advocate multi-omics integration to decode fire-adapted functional gene networks, guiding climate-resilient forest management.

## 1. Introduction

Forest fires are a critical ecological factor in forest ecosystems [1]. In recent decades, the frequency and intensity of extreme forest fires have increased globally, resulting in a significant annual expansion of burned areas. The Daxinganling region of China, a high fire-incidence zone, recorded 1552 fire events between 1965 and 2009 [2], among which the extreme fires of 1987 and 2003 were particularly devastating [3]. The impacts of forest fires on forest ecosystems are multifaceted. On the one hand, moderate fires can reduce forest density, enhancing light availability and nutrient accessibility for vegetation regeneration [4], and also promote seed release from fire-adapted plant species [5], as well as create conditions for fire-resistant microorganisms to reproduce and occupy ecological niches [6] and accelerate organic matter decomposition [7]. On the other hand, the ecological destructiveness of forest fires cannot be ignored. Heavy fires disrupt the microclimate [8], change the physicochemical properties of soil [9], significantly reduce above- and below-ground biomass [10,11,12,13], and cause serious damage to forest ecosystems.

Soil, as a fundamental component of forest ecosystems, plays a pivotal role in post-fire recovery. Soil microorganisms, functioning as primary decomposers, drive 80–90% of soil processes. They facilitate material cycling and energy flow through extracellular enzyme secretion and nutrient assimilation [14,15], making them reliable indicators of soil quality and ecosystem recovery following fire disturbances [16,17]. Forest fires exert complex effects on soil microbial communities through both direct thermal impacts and indirect alterations in soil conditions. Directly, the high temperatures associated with fires can cause mortality of surface-layer microorganisms, particularly heat-sensitive taxa such as fungi and certain bacterial groups [18,19], leading to a marked reduction in microbial abundance and diversity. Indirectly, fires modify soil physicochemical properties and microenvironmental conditions, which subsequently influence microbial survival and metabolic activities [19]. The relationship between soil environment and microbial communities is bidirectional; microorganisms also modulate soil properties, nutrient cycling, enzyme activities, and other ecological functions [20,21]. Therefore, the recovery dynamics of microbial communities are intrinsically linked to the resilience and stability of soil ecosystems.

The Daxinganling region, situated in the cold-temperate zone, is predominantly covered by *Larix gmelinii* forests, which hold significant ecological and economic value [22]. Existing studies on forest fire regimes in this region reveal a burn pattern shaped by the interplay of biogeoclimatic drivers and anthropogenic disturbances [23]. Natural ignition sources are primarily lightning strikes during summer months [24], whereas human-induced fires predominantly occur in autumn within low-elevation zones exhibiting high anthropogenic pressure [25]; post-fire ecosystem recovery exhibits marked spatial heterogeneity: with decades of regeneration required for the recovery of trees, and relatively rapid recovery of shrubs and herbs [26,27]; in terms of soil respiration rate: the intensity of fire significantly affects microbial-driven carbon cycling processes, and heavy fire can lead to a decrease in soil respiration rate and even transform fire sites into carbon sources [22,28]. Although research in the above areas has been systematic, limited attention has been paid to the recovery of soil microbial communities following fire events, particularly with respect to microbial biomass and metabolic capacity during short-term recovery periods. To address this knowledge gap, the present study employs phospholipid fatty acid (PLFA) analysis and Biolog-Eco microplate technology to systematically investigate changes in fungal and bacterial community composition and metabolic capacity. Additionally, the influence of soil physicochemical properties on microbial community assembly post-fire is explored. By elucidating the short-term recovery patterns of soil microbial communities under varying fire intensities, this study aims to provide a scientific foundation for post-fire recovery management in cold-temperate *Larix gmelinii* forest ecosystems and to advance our understanding of soil microbial ecological processes following forest fires.

## 2. Materials and Methods

### 2.1. Sample Site Overview

**Figure 1:**
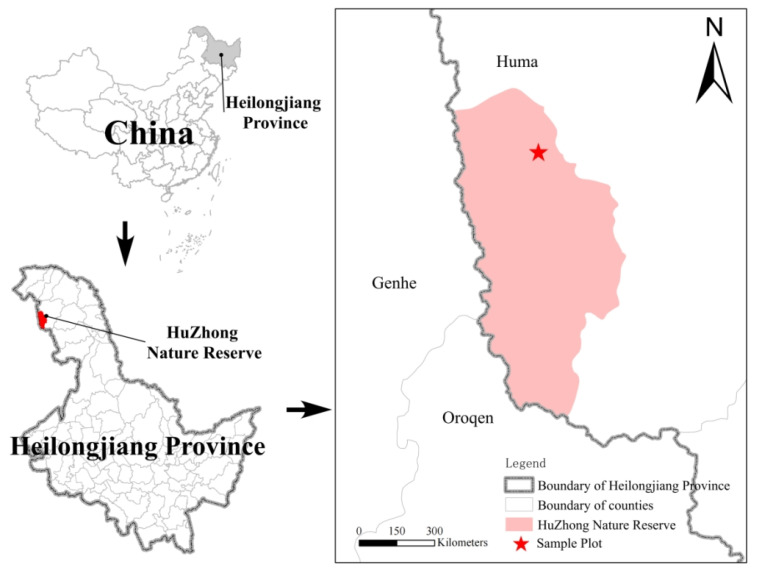
The asterisk indicates the study site in Heilongjiang Province and China.

The investigation was conducted within the Huzhong National Nature Reserve (122°12′16.3′′–122°21′7.8′′ E, 53°26′30.6′′–53°28′6.3′′ N), located in the Daxinganling region of Heilongjiang Province, China (Figure 1). This reserve represents a pristine cold-temperate continental monsoon climate ecosystem, characterized by an annual mean air temperature of −4.0 °C and a mean annual precipitation of 458.3 mm, with approximately 70% of precipitation occurring during summer months (June–August). The frost-free period averages 80–85 days annually. Topographically, the area exhibits an elevation gradient of 800–1200 m above sea level, with dominant soil types including brown coniferous forest soil, meadow soil, swamp soil, and lithosol. This region is recognized as one of China’s most ecologically intact cold-temperate coniferous forest ecosystems.

### 2.2. Experimental Design and Sampling Strategy

In July 2024, four distinct fire-impacted zones were identified within the reserve, corresponding to areas affected by wildfires in 2023. These zones were categorized into three fire severity classes—light (L), moderate (M), and heavy (H)—based on established forest fire intensity classification criteria (Table 1). Unburned control plots (CK) were selected in adjacent undisturbed areas exhibiting comparable elevation (±50 m), vegetation composition, slope gradient (±5°), and aspect (±15°). To minimize spatial autocorrelation and edge effects, seven replicate quadrats (20 m × 20 m) were systematically established within each fire severity class and control area (28 quadrats total). Quadrats were spaced ≥20 m apart and positioned ≥5 m from burn boundaries.

### 2.3. Soil Sampling and Processing

Following the removal of surface litter and humus, soil cores (0–10 cm depth) were collected from each quadrat using a standardized five-point sampling method. Samples were homogenized, sieved (2 mm mesh) to remove coarse roots and gravel, and immediately stored in sterile polyethylene bags within ice-cooled containers to preserve microbial integrity. Subsamples were divided into two portions: one aliquot was frozen at −20 °C for subsequent microbiological analyses, while the remaining soil was air-dried at ambient temperature (25 °C), mechanically ground, and sieved (<0.15 mm) for physicochemical characterization.

### 2.4. Soil Physicochemical Analysis

Soil physicochemical properties were analyzed using standardized protocols. Moisture content (MC) was quantified by oven-drying fresh soil at 105 °C to constant mass [30], while soil pH was measured potentiometrically in a 1:2.5 (*w*/*v*) soil-water suspension [31]. Dissolved organic carbon (DOC) was extracted via ultrapure water leaching (1:10 *w*/*v*), filtered through 0.45 μm membranes, and analyzed using a TOC analyzer (Shimadzu TOC-L, Kyoto, Japan) [32]. Black carbon (BC) content was determined by pyrolysis-oxidation at 375 °C under oxygen-limited conditions [33]. Soil organic carbon (SOC) and total nitrogen (TN) were measured by dry combustion with a CN elemental analyzer (Elementar Vario EL III) (Elementar Analysensysteme GmbH, Langenselbold, Germany) [34]. Available nitrogen fractions were further characterized: alkali-hydrolyzable nitrogen (AN) was extracted with 1.0 M NaOH and quantified by micro-Kjeldahl distillation [35], available phosphorus (AP) was assessed via molybdenum blue spectrophotometry after 0.5 M NaHCO_3_ extraction (pH 8.5) [36], and available potassium (AK) was determined by flame photometry following 1.0 M NH_4_OAc extraction (pH 7.0) [37].

### 2.5. Determination of Soil Enzyme Activities

Soil urease (S-UE) activity was determined by the phenol-sodium hypochlorite colorimetric method [38]. Soil dehydrogenase (S-DHA) activity was determined by the tetrazolium salt reduction method [39]. Soil sucrase (S-SC) activity was determined by the colorimetric method using 3, 5-dinitrosalicylic acid (DNS) [40]. Soil fluorescein diacetate enzyme (S-FDA) was determined spectrophotometrically [41]. Soil acidic protease (S-ACPT) was determined by the ninhydrin colorimetric method [42].

### 2.6. Detection of Phospholipid Fatty Acids

Detection of phospholipid fatty acids (PLFA) was accomplished by methyl esterification of potassium hydroxide-methanol solution [43], quantified by using nineteen alkanoic acid as an internal standard, detected in a meteorological spectrometer TRACE 1300, and analyzed by using the herlock MIS 4. system to analyze the composition of phospholipid fatty acids in the PLFA profiles of soil samples, with the fatty acid concentration expressed in nmol/g. Different PLFAs were used to calibrate specific microorganisms [44,45]. The content of PLFAs in the samples was calculated according to the following equation [46]:

N = [Target Response/(19:0)Response] × (19:0)Concentration × [Dissolved sample volume/Sample dry weight × FAME] (1)

where Equation (1): N stands for fatty acid content (nmol/g), Response is the response value of the biomarker, 19:0 is the internal standard c19:0 (ng/μL), FAME is the molar mass of fatty acid methyl ester (g/mol), Dissolved sample volume is in μL, and sample dry mass is in g.

### 2.7. Determination of Carbon Source Metabolic Activity of Soil Microorganisms

The carbon metabolic activity of soil microorganisms was determined by the Biolog-Eco microplate method as follows: 10 g of fresh soil was weighed and added to 90 mL of sterile saline with a mass fraction of 0.85%, and then the supernatant was shaken and diluted to 10^−3^ times, and then 150 μL of the supernatant was sucked into the wells of the Biolog-Eco plate and incubated at a constant temperature of 25 °C for 384 h. The sample was incubated at a constant temperature of 25 °C for 384 h, and then measured every 24 h with an enzyme marker at a wavelength of 590 nm. The average well color development (AWCD) represents the ability of the microbial community to utilize a single carbon source, i.e., the overall activity of microorganisms, and was calculated as follows:

(2) $$AWCD=\sum \left(Ci-R\right)/n$$

where Equation (2) Ci is the optical density value of each hole of the carbon source, R is the optical density value of the control hole, n is the number of carbon sources, when Ci-R < 0, it is recorded as 0.

### 2.8. Data Analysis

Microbial community alpha diversity was assessed using the Shannon–Wiener index (H), Simpson’s dominance index (D), Margalef’s richness index (M), and Menhinick’s richness index (E) [46]. Functional metabolic diversity was characterized through the Shannon–Wiener (H), McIntosh (U), and Simpson (D) indices. Raw data were compiled in Microsoft Excel 2021, followed by normality (Shapiro–Wilk test) and homogeneity of variance (Levene’s test) verification in SPSS 27.0. Significant differences (*p <* 0.05) in alpha diversity indices, soil physicochemical properties, PLFA biomarkers, and AWCD across fire severity classes were evaluated using one-way ANOVA with Tukey’s post hoc test. Principal component analysis (PCA) of microbial community composition was conducted using the vegan package (v2.6-4) in R (v4.2.1), and environmental drivers were identified through Mantel tests.

## 3. Results

### 3.1. Changes in Soil Physicochemical Properties After Short-Term Restoration of Fire Burn Sites

**Figure 2:**
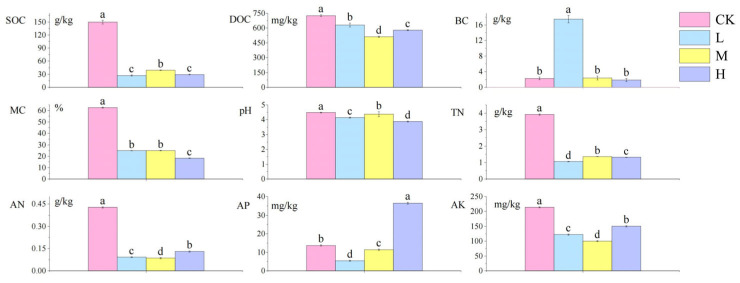
Differences in the content of soil physical and chemical factors after short-term recovery of burned sites with different intensities. All results are reported as mean ± standard deviation (n = 4). Different letters within a row indicate significant differences (*p <* 0.05; ANOVA) among the different intensities of fire in this study. CK, Control—blank; L, light fire; M, moderate fire; H, heavy fire.

The analysis of soil physicochemical properties following short-term recovery in cold-temperate *Larix gmelinii* forest fire-burned sites is presented in Figure 2. Compared to the control group, the fire-burned sites showed significantly lower contents of SOC, DOC, MC, pH, TN, AN, and AK (*p <* 0.05). Notably, the L site exhibited a significant increase in BC (*p <* 0.05), while the H site displayed significantly higher AP content than the control group (*p <* 0.05). Among fire-affected groups, the M site demonstrated significantly elevated SOC, pH, and TN levels compared to other fire groups (*p <* 0.05), whereas DOC, AN, and AK showed contrasting trends with significantly reduced contents (*p <* 0.05).

### 3.2. Changes and Differences in Soil Enzymes

**Figure 3:**
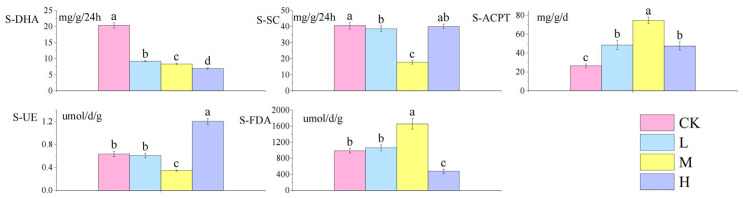
Differences in soil enzyme activities after short-term recovery in burned areas with different intensities. Different letters within a row indicate significant differences (*p <* 0.05; ANOVA) among the different intensities of fire in this study. CK, Control—blank; L, light fire; M, moderate fire; H, heavy fire.

As shown in Figure 3: compared with the control group, S-DHA decreased significantly with the increase in fire intensity (*p <* 0.05), S-SC activity decreased significantly (*p <* 0.05), S-ACPT activity increased significantly (*p <* 0.05), S-UE showed a V-shaped trend of decreasing first and then increasing (*p <* 0.05), and the trend of S-FDA activity was opposite to that of soil urease.

### 3.3. Changes in Soil Microbial Community Composition and Functions

#### 3.3.1. Soil Microbial Community Composition and Biomass Changes

**Figure 4:**
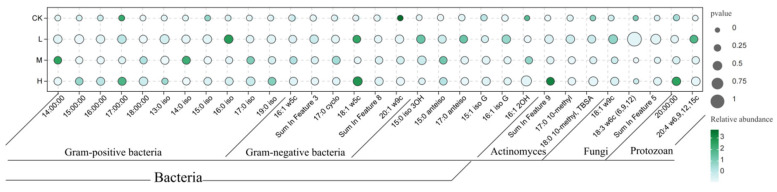
Schematic diagram of the relative abundance of soil microbial phospholipid fatty acids after short-term recovery in burned areas with different intensities.

Phospholipid fatty acid (PLFA) analysis detected 31 biomarkers representing distinct microbial groups (Figure 4), including 11 for Gram-positive bacteria (G^+^), 6 for Gram-negative bacteria (G^−^), 6 for other bacteria, 3 for actinomycetes, 3 for fungi, and 3 for protozoa.

**Figure 5:**
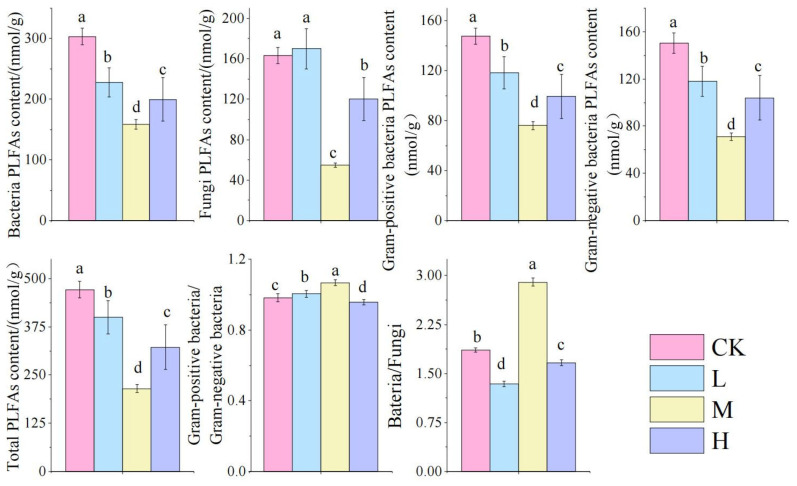
Changes in soil microbial biomasses after short-term recovery in burned areas with different intensities. Different letters within a row indicate significant differences (*p <* 0.05; ANOVA) among the different intensities of fire in this study. CK, Control—blank; L, light fire; M, moderate fire; H, heavy fire.

As shown in Figure 5: Compared to the control group, fire-burned sites exhibited significantly reduced biomass of bacteria, fungi, G^+^, G^−^, and total PLFAs (TPLFA) (*p <* 0.05). These microbial parameters followed a V-shaped pattern across fire intensity gradients, with the M group showing significantly lower values than other fire groups (*p <* 0.05). Notably, the M group demonstrated significantly higher bacterial-to-fungal biomass ratios (Ba/Fu) and Gram-positive to Gram-negative ratios (G^+^/G^−^) compared to other fire-affected groups (*p <* 0.05), indicating fire-driven restructuring of microbial community composition.

#### 3.3.2. Functional Shifts in Microbial Carbon Metabolism

**Figure 6:**
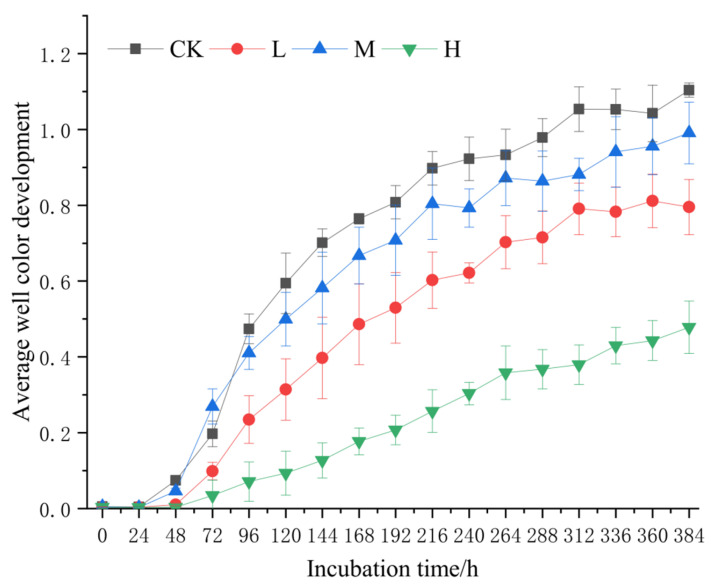
Average color change rate of soil microbial community after short-term recovery of burned sites with different intensities. CK, Control—blank; L, light fire; M, moderate fire; H, heavy fire.

The AWCD curve exhibited triphasic dynamics (Figure 6): microbial activity showed progressive increases from 0 to 24 h, accelerated utilization between 24 and 312 h, and reached metabolic equilibrium after 312 h. Across all timepoints, AWCD values followed the hierarchy CK > M > L > H.

**Figure 7:**
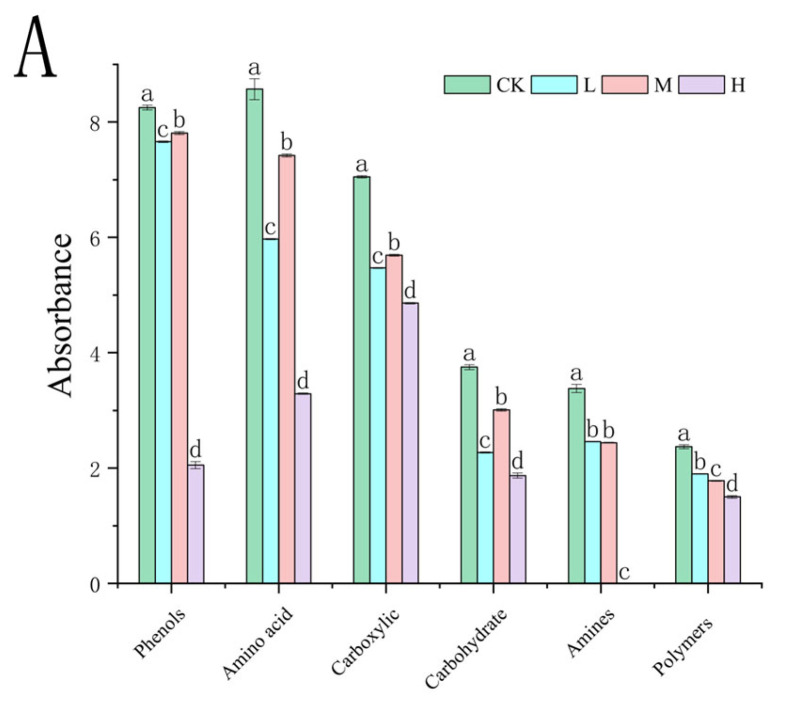

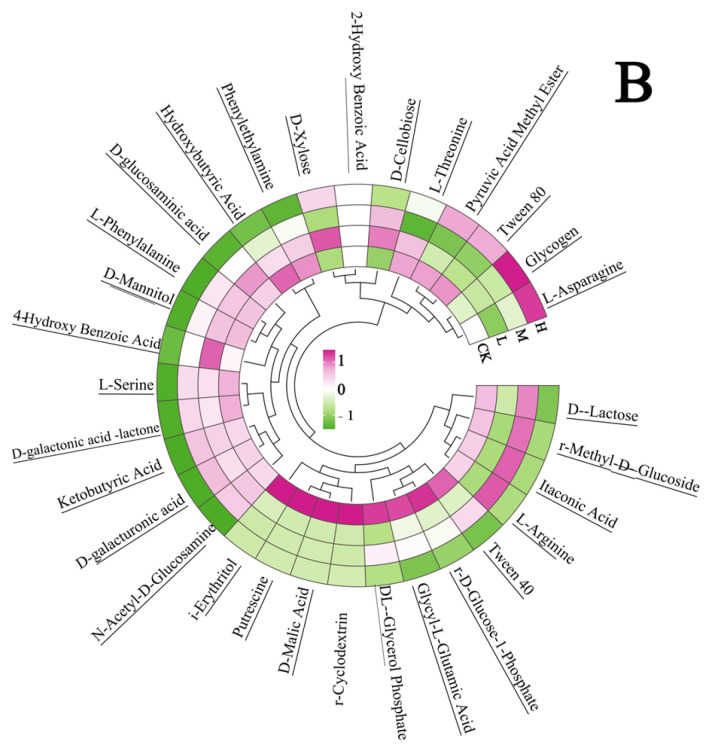
Carbon source utilization efficiency (**A**) and carbon source type (**B**) of the soil microbial community after short-term recovery of burned scars with different intensities. Different letters within a row indicate significant differences (*p <* 0.05; ANOVA) among the different intensities of fire in this study. CK, Control—blank; L, light fire; M, moderate fire; H, heavy fire.

At the 312 h metabolic inflection point (Figure 7A), fire disturbance significantly reduced microbial carbon source utilization capacity compared to controls (*p <* 0.05), with the H group demonstrating more severe impairment than the L and M groups. (*p <* 0.05). Substrate-specific analysis revealed distinct functional adaptations: L group microbes exhibited enhanced utilization of surfactants (Tween 40), phosphorylated compounds (α-D-glucose-1-phosphate, D,L-α-glycerol phosphate), and specialized substrates including α-cyclodextrin, glycyl-L-glutamic acid, putrescine, D-malic acid, and i-erythritol (*p <* 0.05 vs. M/H). The M group displayed preferential metabolism of disaccharides (α-D-lactose, β-methyl-D-glucoside) and specific organic acids (itaconic acid, L-arginine) (*p <* 0.05 vs. L/H). The H group showed selective upregulation in glycogen and L-asparagine utilization (*p <* 0.05 vs. L/M). These fire intensity-dependent metabolic patterns indicate functional reorganization of microbial communities, with differential substrate specialization across burn severity gradients.

#### 3.3.3. Correlation Analysis of Factors Affecting Microbial Community Composition and Functioning

**Figure 8:**
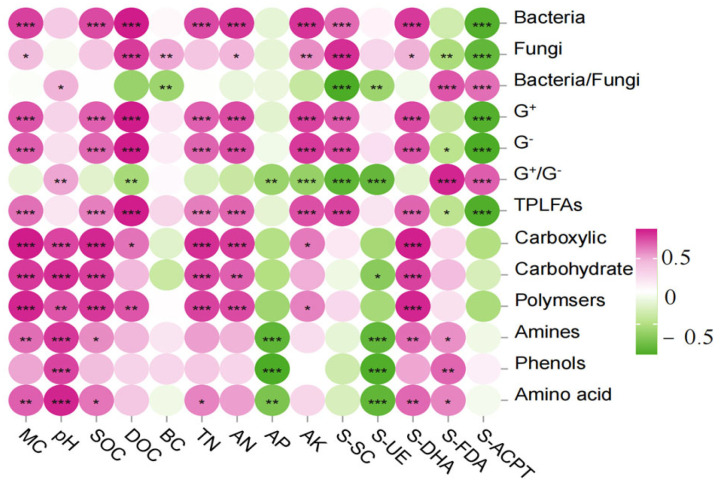
Correlation heat map of soil microbial community composition and function with physicochemical factors and enzymes. Note: *X*-axis represents soil environment and enzymes, *Y*-axis represents species composition and function. The *p*-value is shown in different colors in the figure. If the *p*-value is less than 0.05, it is marked with an * sign, ** 0.01 < *p* ≤ 0.05, *** 0.001 < *p* ≤ 0.01.

The correlation analysis between the content of PLFAs and physicochemical factors in the soil of the burnt site (Figure 8) showed that the content of bacteria, G^+^, G^−^, and total PLFAs were highly significantly positively correlated with DOC, MC, SOC, DOC, TN, AN, AK, S-SC, and S-DHA (*p <* 0.01) and highly significantly negatively correlated with S-ACPT (*p <* 0.01); the correlation analysis between Fu and DOC, BC, AK, and S-SC were highly significant positively correlated (*p <* 0.01) and highly significant negatively correlated (*p <* 0.01) with S-FDA, S-ACPT; G^+^/G^−^ and Ba/Fu were highly significant positively correlated (*p <* 0.01) with S-FDA, S-ACPT and highly significant negatively correlated (*p <* 0.01) with S-SC, S-UE.

The correlation analysis between the ability of soil microorganisms to utilize the six types of carbon sources in the fire-scorched site and the physicochemical factors showed that carboxylic, Carbohydrate, and Polymers were highly significantly and positively correlated with MC, pH, SOC, TN, AN, and S-DHA (*p <* 0.01); Amino and Amino acid were highly significantly and positively correlated with MC, PH, and S-DHA (*p <* 0.01); Amino and Amino acid were highly significantly and negatively correlated with S-SC, and S-UE (*p <* 0.01); and S-SC and S-UE (*p <* 0.01). Amino and Amino acid were highly significantly positively correlated with MC, PH, S-DHA (*p <* 0.01), SOC, S-FDA (*p <* 0.05), and AP, S-UE (*p <* 0.01); phenols were highly significant positively correlated with pH, S-FDA (*p <* 0.01), and AP, S-UE (*p <* 0.01); DOC was highly significant positively correlated with polymers (*p <* 0.01); DOC was highly significant positively correlated with polymers (*p <* 0.01). significantly positively correlated with polymers (*p <* 0.01), and significantly positively correlated with carboxylic (*p <* 0.01).

### 3.4. Differences in Soil Microbial Communities and Functional Diversity in Fire-Scarred Sites

#### 3.4.1. Alpha Diversity

**Figure 9:**
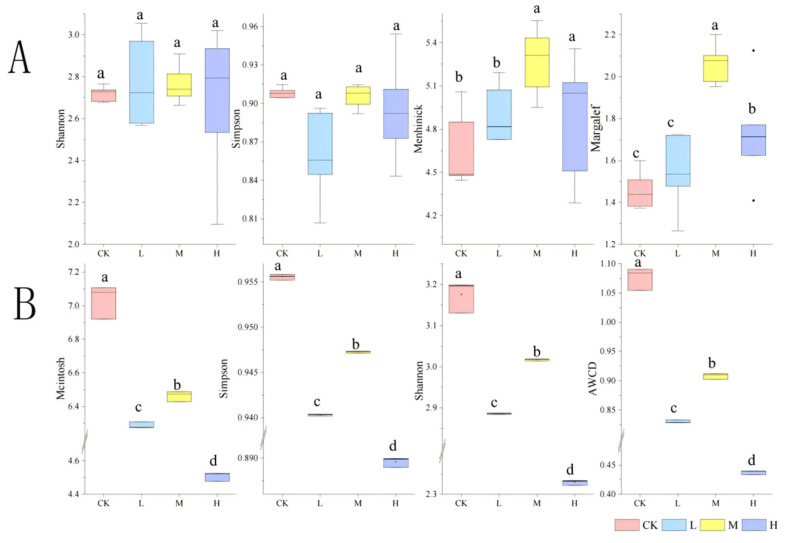
Alpha diversity of soil microbial composition (**A**) and function (**B**) after short-term recovery of burned sites with different intensities.CK, Control—blank; L, light fire; M, moderate fire; H, heavy fire. Different letters within a row indicate significant differences (*p <* 0.05; ANOVA) among the different intensities of fire in this study. CK, Control—blank; L, light fire; M, moderate fire; H, heavy fire.

The alpha diversity analysis revealed differential recovery patterns among fire-impacted groups (Figure 9A). Short-term restoration (1 year post-fire) showed no significant differences in Shannon and Simpson indices between burned sites (L, M, H) and the control (CK) (*p* > 0.05), indicating preserved community evenness. The Margalef and Menhinick indices of group M were significantly higher than those of group H (*p <* 0.05), suggesting enhanced species richness under moderate fire intensity. Specifically, group L restored microbial community diversity to pre-fire levels, while group M increased diversity, and group H significantly elevated taxonomic richness.

As delineated in Figure 9B, fire disturbance significantly reduced all functional diversity indices compared to the control group (*p <* 0.05). However, hierarchical recovery patterns emerged within fire-affected groups: group M exhibited significantly higher functional diversity than both L and H groups (*p <* 0.05).

#### 3.4.2. Drivers of Alpha Diversity

**Figure 10:**
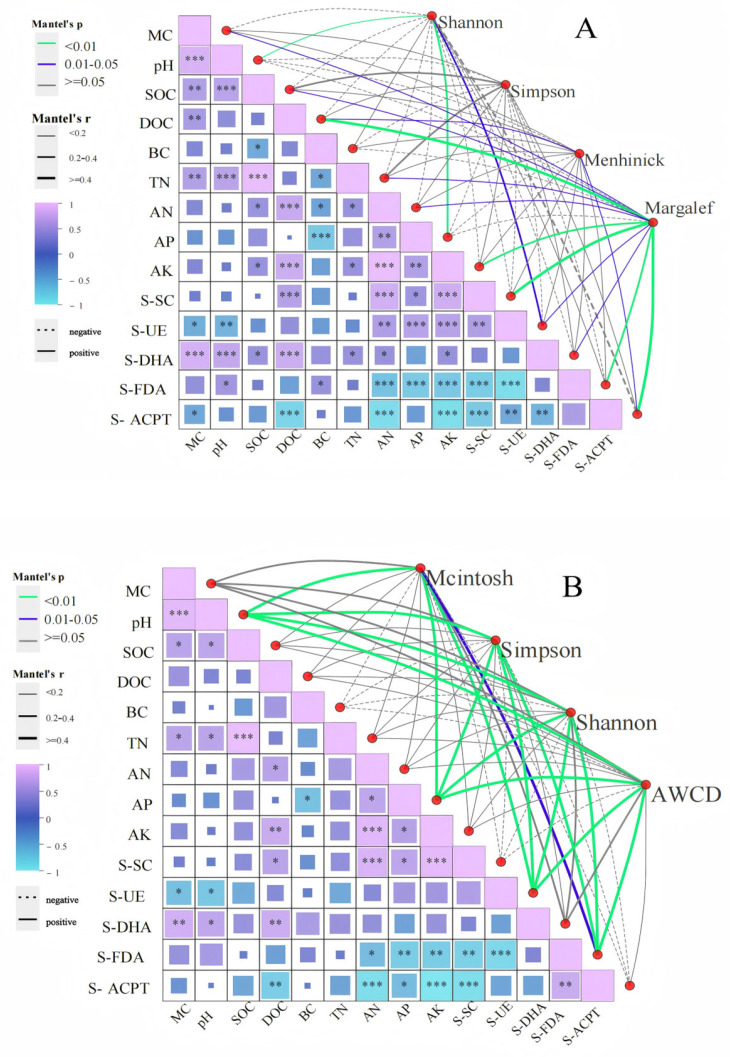
Alpha diversity and physicochemical correlation between soil microbial composition (**A**) and function (**B**) after short-term recovery of burned sites with different intensities. If the *p*-value is less than 0.05, it is marked with an * sign, ** 0.01 < *p* ≤ 0.05, *** 0.001 < *p* ≤ 0.01.

Correlation analysis revealed distinct environmental linkages shaping microbial alpha diversity (Figure 10A). The Shannon index exhibited strong positive correlations with pH and AP (*p <* 0.01), while the Menhinick index was positively associated with DOC and S-ACPT (*p <* 0.01). The Margalef index demonstrated significant positive relationships with DOC, AK, S-SC, S-FDA, and S-ACPT (*p <* 0.01).

Functional correlations further highlighted systemic interactions (Figure 10B): Simpson’s, Shannon’s, and AWCD indices were strongly linked to pH, AP, S-UE, and S-FDA (*p <* 0.01), whereas the McIntosh index showed a positive association with S-FDA (*p <* 0.05).

#### 3.4.3. Beta Diversity Patterns

**Figure 11:**
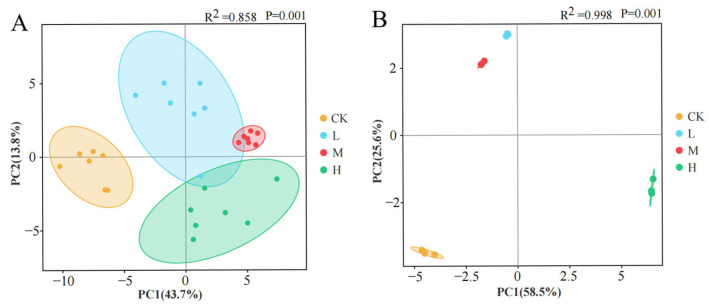
Principal component analysis of soil microbial community composition (**A**) and carbon source utilization capacity (**B**) after short-term recovery of burned scars with different intensities.

PCA revealed fire-driven divergence in microbial community composition and function (Figure 11). Compositional PCA (Figure 11A) demonstrated 57.5% cumulative variance explanation (PC1:43.7%, PC2:13.8%), while functional PCA (Figure 11B) accounted for 84.1% total variance (PC1:58.5%, PC2:25.6%). Spatial distribution patterns showed distinct clustering: L and M groups occupied adjacent positions in the second quadrant, contrasting with unburned controls in the third quadrant and the H group in the fourth quadrant. This quadrant-specific segregation indicates significant fire intensity-dependent restructuring of both microbial community architecture and metabolic functional profiles.

## 4. Discussion

### 4.1. Post-Fire Dynamics of Soil Physicochemical Properties

As a critical ecological driver in forest ecosystems, fire disturbance exerts profound impacts on soil nutrient cycling. Our findings demonstrate significant reductions in SOC and DOC content across fire-affected sites relative to unburned controls, primarily attributable to pyrolytic carbon loss and post-fire leaching processes. Combustion of aboveground biomass and humus layers during burning directly mineralizes surface carbon stocks through gaseous emissions (CO_2_/CH_4_), constituting the primary depletion pathway [47,48]. Secondly, the fire burned the surface vegetation material to varying degrees, resulting in a significant reduction in the vegetation cover of the fire burned site, and even the surface was bare, and the reduction in the vegetation material not only weakened the water-holding capacity of the soil, but also increased the risk of the soil to be eroded by rainfall washout and runoff. The intensified leaching further accelerated the loss of SOC and DOC, leading to a continuous reduction in the soil carbon pool [49]. Notably, moderate-intensity burns exhibited paradoxical SOC enrichment compared to light/heavy-intensity treatments. This anomaly likely stems from fire-generated canopy gaps that enhance photosynthetically active radiation (PAR) and soil temperature, stimulating understory revegetation. Accelerated root exudation and litter input under these conditions facilitate organic matter accumulation, offsetting initial combustion losses [50]. Having more forest windows formed after canopy burning than the light fire site, and also having better vegetation conditions than the heavy fire site, the moderate fire site thus creates an optimal “regeneration window” balancing carbon loss and renewal mechanisms.

Fire disturbance induces significant reductions in soil moisture content (MC), a finding consistent with prior studies by Jian-jian Kong et al. [51]. This decline arises from direct and indirect mechanisms linked to fire severity and post-fire landscape alterations. Direct effects include the thermal mortality of vegetation, which eliminates canopy shading and disrupts the vegetation-soil water retention system, thereby accelerating surface evaporation and depleting soil moisture reserves. Concurrently, vegetation loss diminishes root-mediated water uptake and storage, destabilizing hydrological equilibrium. Indirect effects are associated with three post-fire landscape alterations: bare ground surfaces [52], expanded forest canopy gaps [53], and black ash deposition [54]. The latter, a hallmark of post-fire ground cover, reduces surface albedo, enhancing solar radiation absorption and elevating soil temperatures. This thermal amplification accelerates soil moisture evaporation while altering soil microclimates (e.g., vapor pressure gradients, thermal conductivity), thereby disrupting the equilibrium of moisture retention and redistribution dynamics.

Contrary to conventional observations of post-fire pH elevation via base cation release [55,56,57], our study documented soil acidification. This anomaly arises from two counteracting mechanisms: (1) leaching-driven migration of fire-generated alkaline oxides (Ca^2+^, Mg^2^, K^+^) to subsurface horizons, depleting surface buffering capacity; (2) enhanced organic acid production (humic/fulvic acids) from charred litter decomposition and root exudates during early revegetation, lowering surface pH [58]. Nitrogen dynamics exhibited parallel depletion trends, with TN and AN reduction aligning with pyrolysis-induced volatilization [59,60,61] and microbial immobilization during secondary succession [62]. Notably, AP increased substantially in heavy-severity burns, consistent with Fernández-García and Moya et al. [63,64]. Intense fires mineralized the thick organic horizon of Daxing’anling forests, depositing ash-rich, labile phosphorus onto bare soil surfaces. In contrast, light/moderate burns exhibited lower AP, AN, and AK due to incomplete organic matter combustion and preserved soil-litter interfaces that limited nutrient release.

### 4.2. Response of Soil Microbial Biomass After Short-Term Restoration of Fire Burn Sites

Soil microbial biomass, a critical indicator of microbial community activity and functionality, exhibits dynamic responses to fire disturbance that reflect short-term ecosystem impacts [65]. This study demonstrated significant post-fire reductions in microbial biomass, primarily attributed to thermal radiation exceeding microbial lethal thresholds during combustion, directly causing mortality [66]. Despite one year of recovery, biomass failed to rebound to pre-fire levels, aligning with the multiple stressor hypothesis: fire-induced alterations in soil nutrients (e.g., AN, AK), pH, moisture, and enzymatic activity collectively impair microbial viability and metabolic capacity [67].

Notably, microbial biomass correlated positively with MC, DOC, AN, and AK. DOC serves as a primary microbial carbon substrate, with its availability directly regulating microbial growth [68]. However, fire-driven declines in labile nutrients (AN, AK) disrupted nutrient cycling, constraining biomass recovery. Paradoxically, elevated AP in heavy-severity burns did not enhance biomass, likely due to synergistic limitations: (1) severe organic matter loss and structural degradation reduced microbial access to AP; and (2) acidic conditions promoted AP fixation as insoluble Fe/Al-phosphates, rendering it biologically inaccessible despite increased concentrations [69].

S-DHA, a critical biomarker of microbial activity [70], exhibited sustained suppression in fire-affected sites compared to unburned controls even after one year of recovery. The decline in S-DHA activity showed significant positive correlations with MC, pH, SOC, DOC, TN, AN, and AK, highlighting nutrient co-limitation as a key driver of microbial metabolic constraints [67]. The low DOC content in the soil of the moderate fire site suggests that its SOC may be dominated by difficult-to-degrade fractions. This inference is further supported by the results of PLFA analyses in the later section: the soil microbial community in the moderate fire site showed significant oligotrophic characteristics (G^+^/G^−^, Ba/Fu rising). Thus, although total SOC was significantly higher in the moderate fire site than in the light and heavy fire sites, S-DHA activity did not peak here due to the decrease in microbially available substrates resulting from the increased chemical stability of the carbon fraction. The thermal sensitivity of enzymatic proteins explains the initial activity loss, as combustion directly disrupts protein conformation and catalytic functionality [71]. Fire disturbance further impaired enzymatic resilience through dual pathways: direct thermal inactivation and indirect substrate limitation caused by reduced organic carbon inputs from vegetation loss, thereby diminishing hydrolytic enzyme activity (e.g., S-SC) [72]. Importantly, enzymatic recovery followed time-dependent succession patterns, requiring multiple growth cycles to restore pre-fire catalytic capacity through microbial community reorganization and organic matter reaccumulation [71].

The biomass of soil bacteria and fungi was significantly lower in moderate fire sites than in light and heavy fire sites, and moderate-intensity fires significantly increased microbial stress indices (G^+^/G^−^ and Ba/Fu ratios) compared to controls. Elevated G^+^/G^−^ ratios indicate heightened environmental nutrient stress favoring oligotrophic Gram-positive bacteria [73], while increased Ba/Fu reflects reduced ecosystem resilience due to disproportionate fungal suppression [74]. These shifts suggest moderate burns induce vulnerable soil ecosystems with microbial communities transitioning from r-strategy to k-strategy. The pronounced Ba/Fu elevation in moderate burns aligns with fungal thermal sensitivity [75,76], whereas Ba/Fu reduction in light/heavy burns involves distinct mechanisms: in this study, we found a significant increase in soil black carbon in lightly burned sites, with black carbon shown to enhance soil aeration [77]. Consequently, light-intensity fires promoted the dominance of fungi over anaerobic bacteria [78], while sustained acidification in heavily burned soils enhances fungal competitiveness under low pH conditions [79]. Such intensity-dependent dynamics highlight how fire severity differentially regulates microbial guilds through thermal mortality and physicochemical habitat modification, with moderate burns exacerbating nutrient stress and light/heavy burns altering microbial community biomass via microenvironmental shifts.

### 4.3. Functional Resilience of Soil Microbial Communities Post-Fire

The AWCD index, a critical metric for assessing microbial metabolic activity, demonstrated fire intensity-dependent suppression across burned sites [80,81]. In this study, the main carbon sources utilized by soil microorganisms in burned and unburned plots were phenols, Amino acids, carboxylic acids, and carbohydrates. The utilization patterns of these four carbon sources were consistent with trends in the overall metabolic activity of microorganisms under different fire intensities, which followed the following hierarchy: M > L > H burns, reflecting differential functional recovery trajectories. This suppression stems from coupled biotic and abiotic constraints: reduced microbial biomass (PLFA analysis) directly limits catalytic potential, while nutrient depletion imposes secondary metabolic stress [82]. Heavy-intensity burns exerted pronounced sterilization effects [83,84], drastically reducing surviving microbial inocula. Concurrently, moisture deficits in heavily burned soils exacerbated physiological constraints on residual communities. The superior metabolic performance in moderate burns likely arises from intermediate disturbance effects [85], where partial canopy removal enhances light/temperature regimes without inducing extreme microclimatic shifts. This “optimal stress” window facilitates microbial community reassembly through competitive exclusion of sensitive taxa while maintaining sufficient resource availability. Enhanced forest gap dynamics in moderate burns may further stimulate phototroph-driven carbon cycling, indirectly boosting heterotrophic activity through substrate provisioning.

The metabolic capacity of soil microorganisms, particularly their utilization of six carbon source types, exhibited significant positive correlations with MC and pH. Soil moisture deficits directly impair microbial physiological status by disrupting osmotic balance and membrane integrity [86], while indirectly limiting carbon substrate supply through reduced root exudation from fire-suppressed vegetation [87]. The incomplete recovery of MC in burned sites likely perpetuates metabolic constraints by sustaining water stress. Concurrently, pH-mediated effects on microbial activity align with acidification-induced metabolic suppression [88], where energy diversion toward intracellular pH homeostasis reduces substrate conversion efficiency.

Enhanced S-FDA activity in moderate burns paralleled AWCD trends, corroborating the intermediate disturbance hypothesis through intensified nutrient transformation. The positive S-FDA/S-ACPT correlation reflects acid-driven proteolytic dynamics: S-ACPT-mediated protein hydrolysis generates bioavailable peptides that fuel microbial metabolism, elevating S-FDA activity [89]. Notably, the negative correlation between S-ACPT and soil nutrients may in fact reflect an adaptive response triggered by nutrient loss following fire: microorganisms invest in enzyme production when they are nutrient-limited, typifying the resource trade-offs in allocation theory. By dynamically regulating enzyme synthesis, microbes optimize resource allocation in response to environmental stresses to ensure survival and reproductive success [90]. Environmental stresses also activate the rhizosphere priming effect (RPE) by enhancing the release of root exudates (e.g., soluble sugars, organic acids). These exudates serve as fast-acting carbon sources for microbial metabolism, stimulating microbes to synthesize extracellular enzymes that accelerate organic matter decomposition and facilitate the acquisition of scarce nutrient resources [91].

### 4.4. Post-Fire Dynamics of Soil Microbial Diversity and Functionality

The results of this study showed that after one year of recovery in the fire-affected area, there was no significant change in the Shannon and Simpson indexes of plfa markers in the burned areas, indicating that the overall diversity of soil microorganisms had recovered to the level before the fire. In addition, the study found that the Menhinick and Margalef indices, which characterize richness, were significantly increased. This may be due to fire acting as a filtering factor, removing some heat-sensitive microbial groups, living vacated niches are colonized by heat-resistant survivors, adjacent migrants, and pioneer taxa [92,93]. The richness of plfa markers peaked at moderately burned sites, consistent with the moderate disturbance hypothesis, which posits that moderate disturbance disrupts the dominance of dominant species, thereby promoting the highest level of diversity and facilitating the invasion and colonization of additional species [94]. However, functional diversity of soil microorganisms in the fire-affected area exhibited an overall declines, likely due to two primary mechanisms: (1) Biochemical constraints imposed by fire-induced organic matter depletion and persistent water deficit, which directly restrict microbial metabolic activity and reduce the repertoire of functional traits; (2) Environmental filtering under harsh conditions, which enriches stress-tolerant taxa that predominantly belong to functionally homogenized groups. These processes collectively drive community functions toward simplification [95].

This study found significant positive correlations between soil pH and both the composition and functional diversity of microbial communities, which is in line with previous studies that concluded that pH is an important driver of microbial communities [96,97,98]. This may be attributed to pH indirectly shaping microbial community composition by influencing multiple environmental factors, such as soil nutrient availability and soil enzyme activity. For instance, acidic conditions inhibit taxa mediating nitrogen fixation, sulfur oxidation, and phosphorus solubilization—key processes requiring neutral to alkaline microenvironments for optimal activity [99]. Post-fire pH shifts likely amplify these constraints, creating important filters that suppress acid-sensitive functional guilds while favoring stress-tolerant taxa. Therefore, changes in soil pH following a fire represent one of the critical factors influencing the recovery of microbial community and functional diversity.

## 5. Conclusions

Fire intensity modulated soil biogeochemical cycling, driving significant reductions in SOC, DOC, AN, AK, and TN. After one year of post-fire recovery, microbial biomass and metabolic activity in burned areas failed to recover to pre-fire levels. In the moderate fire site, bacterial and fungal biomass exhibited the lowest values, while metabolic activity reached its peak. Although fire increased the richness of PLFA (phospholipid fatty acid) markers, it caused a notable decline in functional alpha diversity and significantly altered both composition- and function-based beta diversity. Future research should prioritize investigating long-term microbial-nutrient coupling mechanisms and employ metagenomic techniques to analyze adaptive responses of key functional genes to post-fire organic matter dynamics, thereby refining the framework for predicting the resilience of cold temperate *Larix gmelinii* forest under escalating fire regimes.

## Figures and Tables

**Table 1:** Basic situation of burning sites with different intensities [29].

Fire Intensity	Grade Index (k·Wm^−1^)	Victimization of Standing Timber	Criteria for Division
L (Light-fire)	350–750	<30%	The understory shrubs are partially burned (less than 50%), and the trunks are blackened at a height of less than 2 m.
M (Moderate-fire)	750–3500	30–70%	The litter layer and the semi-rotten layer are burned, and the color below the semi-rot layer remains unchanged.
H (Heavy-fire)	>3500	>70%	The understory shrubs were all burned and blackened at a height of more than 5 m.

## Data Availability

Data are contained within the article.
